# Elemental Profiling of Fig (*Ficus carica* L.) Cultivars: Nutritional Contribution and Dietary Risk Assessment

**DOI:** 10.3390/foods15071192

**Published:** 2026-04-01

**Authors:** Jiapan Xue, Xiwen Chen, Yixuan Lin, Ziting Chen, Zihan Guo, Hadir Yishake, Ming Wang, Hao Zhang, Jie Yan

**Affiliations:** 1 Key Laboratory of Xinjiang Phytomedicine Resource and Utilization of Ministry of Education, Xinjiang Production and Construction Corps Key Laboratory of Oasis Town and Mountain-Basin System Ecology, College of Life Sciences, Shihezi University, Shihezi 832000, China; 2 Xinjiang Uygur Autonomous Region Academy of Forestry Sciences, Urumqi 830092, China

**Keywords:** fruit, figs, nutrition, heavy metals, ICP-MS

## Abstract

This study analyzed the elemental composition of 20 cultivated fig (*Ficus carica* L.) cultivars, evaluated their contribution to the Recommended Nutrient Intake (RNI), and assessed potential dietary risks associated with trace elements. Thirteen elements (K, Mg, Ca, P, S, Zn, Cu, Mn, Se, B, As, Pb, Cd) were quantified using inductively coupled plasma mass spectrometry (ICP-MS), and the Health Risk Index (HRI) was calculated for trace elements. The results indicated that K was the most abundant mineral, ranging from 197 to 355 mg/100 g fresh weight (FW), followed by P (18–35 mg/100 g FW) and Mg (14–29 mg/100 g FW). A 100 g FW serving provided 9.9–17.8% of the adult RNI for K, 4.2–8.8% for Mg, and 2.5–9.9% for Cu. Multivariate analysis revealed distinct differences in mineral composition among the cultivars, classifying them into four groups. Varieties in Cluster 1 were rich in Mg, Ca, and Zn, whereas those in Cluster 2 exhibited higher Cu content. These findings highlight substantial cultivar-dependent differences in elemental composition and suggest that figs can contribute useful amounts of several essential minerals. In this adult dietary exposure assessment, detected trace element concentrations suggested negligible human health risks based on established experimental conditions. However, the present evaluation did not account for variable mineral bioavailability and individual differences in human intestinal absorption efficiency.

## 1. Introduction

The fig (*Ficus carica* L.), a member of the Moraceae family and the *Ficus* genus, is characterized by its distinctive syconium structure, which arises from the development of numerous small flowers encased within a fleshy receptacle—a notable feature in plant morphology [1,2]. Contemporary nutritional research has identified figs as a rich source of dietary fiber, vitamins, polysaccharides, phenolic compounds, and essential mineral elements. This composition underscores their notable nutritional value and potential health benefits, as supported by existing nutritional studies on edible fruits [3,4,5]. These bioactive constituents not only contribute to the fig’s widespread consumption as a traditional food but also position it as a valuable raw material for the development of functional foods, with promising applications in antioxidation, anti-inflammation, and blood sugar regulation [6,7]. Concurrently, the global fig industry is experiencing expansion. Data from the Helgi Library database indicate a 14.9% increase in global annual fig production in 2022 compared to a decade earlier, with market demand steadily rising. This trend underscores the importance and urgency of systematically evaluating the quality, safety, and nutritional value of figs [8]. Consumption habits of figs vary considerably across regions. In Mediterranean countries, figs are commonly eaten as part of the daily diet, whereas in China, they are more often consumed as seasonal fresh fruit or used in baked goods and processed products. Although the overall daily intake in China remains relatively low, the consumption scenarios have been expanding in recent years. Therefore, evaluating the nutritional contribution and potential risks associated with mineral elements in figs remains meaningful.

At the physiological level, trace elements constitute a fundamental nutritional basis essential for normal growth, development, and metabolic regulation of plants [9,10]. These elements are integral to a myriad of biochemical processes within plants, including photosynthesis, enzyme activity regulation, osmotic pressure maintenance, and the synthesis of secondary metabolites, thereby exerting a comprehensive influence on plant growth [11,12,13,14]. In the context of fruit tree cultivation, the mineral nutritional status critically impacts fruit development, influencing fruit appearance, internal quality, and flavor characteristics [15]. For instance, potassium plays a role in sugar accumulation within the fruit, calcium is involved in cell wall formation affecting fruit firmness, and magnesium, as a core component of chlorophyll, is crucial for the synthesis of photosynthetic products [16,17,18]. A review of the current literature on fig quality research indicates that the primary focus has been on conventional indicators, including soluble solids content, total phenols, polysaccharides, and antioxidant capacity [19]. The available investigations exhibit limited systematic analysis of mineral element composition, particularly regarding comparative profiling of mineral variations among diverse fig germplasms and cultivars. Consequently, undertaking a comprehensive evaluation of the mineral element content in diverse fig varieties is meaningful and necessary for complementing current research gaps. Such an evaluation not only elucidates the varietal differences but also provides a scientific foundation for understanding the intrinsic relationship between mineral elements and fruit quality. This understanding is valuable for informing the breeding of high-quality fig varieties with improved nutritional profiles.

From a human nutrition and health perspective, trace elements are essential micronutrients crucial for maintaining physiological functions and metabolic homeostasis in the human body [20]. They are involved in various physiological processes, including nerve conduction, enzyme-catalyzed reactions, bone development, and immune regulation. Insufficient intake of these mineral elements can result in several health issues, such as selenium deficiency, which diminishes the body’s antioxidant capacity [21]; inadequate potassium levels, which elevate the risk of cardiovascular disease [22]; and magnesium deficiency, which is associated with metabolic disorders [23]. However, it is also important to recognize that excessive intake of certain trace elements poses health risks. For instance, an overabundance of zinc and manganese can lead to digestive discomfort and even liver and kidney damage [24]. Furthermore, with the intensification of environmental pollution, the accumulation of potentially harmful heavy metals in fruits, such as cadmium, lead, and arsenic, has become a significant food safety concern [25]. These toxic elements accumulate through the soil–plant system, and prolonged ingestion may result in neurotoxicity, kidney damage, and even carcinogenic effects. Consequently, a thorough evaluation of the nutritional contributions and potential risks associated with mineral elements in various fig varieties holds significant practical value [26]. This assessment aids in guiding consumers towards informed decision-making and establishing scientifically based intake levels. Furthermore, it provides a theoretical foundation for the development of functional fig products.

Drawing upon the aforementioned context, this study systematically investigated 20 cultivated fig varieties, employing high-precision inductively coupled plasma mass spectrometry (ICP-MS) to quantitatively analyze 13 elements within the fruits. These elements were deliberately selected based on two critical dimensions: nutritional quality evaluation and food safety risk assessment. Specifically, 10 essential mineral elements including K, Mg, Ca, P, S, Zn, Cu, Mn, Se, and B were chosen for their vital roles in fig growth, fruit quality formation, and human dietary nutrition, representing conventional indicators for fruit mineral profiling. Additionally, three priority toxic elements (As, Cd, and Pb) were included as they are the most concerning hazardous metals in agricultural products and are routinely monitored for dietary risk assessment. The primary objective was to comprehensively assess the mineral nutritional value and safety across different varieties. To achieve this, significant difference analysis was utilized to compare inter-varietal differences, while multivariate statistical methods, including hierarchical clustering analysis (HCA) and principal component analysis (PCA), were employed to quantify variations in mineral composition and identify varieties with superior nutritional characteristics. Unlike previous studies on dried figs [27], our research provides a novel contribution by focusing on 20 fresh fig cultivars representative of global diversity. Fresh fruits are nutritionally more relevant for daily consumption, while food processing may alter their mineral composition. By integrating both recommended nutrient intake (RNI) and health risk index (HRI) evaluations, this work delivers a more comprehensive assessment of varietal differences, offering valuable insights for dietary guidance and cultivar selection.

## 2. Materials and Methods

### 2.1. Plant Material

Fruit samples were collected in October 2024 from the Germplasm Repository of Fig located in Urumqi, Xinjiang, China (GPS: 87.51° E, 43.47° N). The site features a temperate continental climate with annual precipitation of approximately 200 mm and sandy loam soil with a pH of 7.5–8.0, typical of the region’s fig cultivation conditions. A total of 20 cultivars were utilized for the study (Supplementary Materials Figure S1): Orphan (OR), White Marseilles (WM), Golden Riverside (GR), Stella (ST), Qingpi (QP), Panache (PN), Adriatic (AD), Bourjasotte Grise (BG), Conadria (CD), Violette Solise (VS), Cherry Tran cddt (CT), Masui Dauphine (MD), Black Mission (BM), Boji Red (BR), Violette de Bordeaux (VB), Adam (AM), De Tres Esplets (TE), Browns Wick (BW), Longue d’Aout (LA), and Schar Amber (SA). The selection of 20 fig cultivars was guided by their global and regional agronomic and commercial prominence. For instance, Conadria, Adriatic, Black Mission, and Brown Turkey are recognized as principal cultivars in California’s fig industry [28]. For each cultivar, 3 to 5 healthy fruit-bearing trees (≥5 years old) were randomly selected as biological replicates, depending on cultivar availability in the germplasm repository. From each tree, at least five mature fruits were harvested and pooled to obtain one composite sample per tree. Each composite sample was homogenized and used for digestion and ICP-MS determination. Therefore, the tree-level composite sample was considered the biological experimental unit in subsequent statistical analyses. Results are presented as mean ± standard deviation (SD) of biological replicates (*n* = 3–5 trees per cultivar). These samples were homogenized using a tissue homogenizer, flash-frozen in liquid nitrogen, and subsequently stored at −80 °C for further analysis.

### 2.2. Elements Analysis via ICP-MS

The selection of the 13 target elements was based on a dual-criteria framework encompassing nutritional significance and food safety concerns. Ten essential elements (K, Mg, Ca, P, S, Zn, Cu, Mn, Se, B) were selected as key indicators for evaluating the nutritional value of figs, as they are essential for plant physiological metabolism and human health. The remaining three elements (As, Cd, Pb) are representative priority toxic heavy metals, which are universally monitored in fruits and foods to assess potential health risks to consumers. All thirteen determined elements, covering both essential nutritional minerals (K, Mg, Ca, P, S, Zn, Cu, Mn, Se, B) and three priority toxic heavy metals (Cd, Pb, As), were quantified simultaneously in one unified ICP-MS analytical workflow, instead of implementing independent detection systems for nutrient elements and heavy metals separately. Homogenized fig fruit samples (0.3–0.4 g) were weighed with a precision of 0.001 g and transferred into a polytetrafluoroethylene (PTFE) digestion vessel. A total of 9 mL of 68% nitric acid (HNO_3_, GR grade, Merck, Darmstadt, Germany) was added, the vessel was covered, and the sample was allowed to stand for one hour. The vessel lid was then secured and digestion was conducted in accordance with the standard operating procedure for the microwave digestion instrument (Mars 6, CEM, Matthews, NC, USA). The digestion protocol comprised three stages: the initial stage at 120 °C with a ramp time of 5 min and a hold time of 5 min; the second stage at 150 °C with a ramp time of 5 min and a hold time of 10 min; and the final stage at 190 °C with a ramp time of 5 min and a hold time of 50 min. Following digestion, the vessel was allowed to cool before cautiously opening the lid to release pressure. The inner lid was rinsed with a small quantity of 2% HNO_3_. Subsequently, the digestion vessel was placed in an ultrasonic bath and ultrasonic degassing was performed for 5 min. Finally, the solution was diluted to 50 mL with 2% HNO_3_, mixed thoroughly, and prepared for analysis, and parallel reagent blank experiments were strictly conducted throughout the whole procedure.

#### Method Validation and Quality Control

The Agilent 8800 ICP-MS/MS was applied for the quantification of 13 target elements. Multi-element standard solutions were adopted for calibration, and all correlation coefficients exceeded 0.9950 (Table S1). The LOD and LOQ were calculated based on 3- and 10-fold standard deviations of blank signals (Table S2). Specific collision/reaction cell modes were optimized to eliminate spectral interference. Strict in-run quality control included continuous calibration verification, reagent blank monitoring, and duplicate sample analyses.

Since no certified reference material (CRM) of fruit matrix was available in the present work, method accuracy was validated via matrix spike recovery tests using fresh fig samples. Obtained recoveries ranged from 85% to 115%, satisfying the routine analytical tolerance for trace element determination in food matrices. The absence of CRMs or inter-laboratory comparison is acknowledged as a limitation, as it restricts full traceability of the measurements.

### 2.3. Mineral Contribution of Figs to RNI

According to Equation (1), assuming an intake of 100 g of fig per day [29], the ratio of each mineral nutrient element in figs to the RNI was calculated as follows:

(1) $$\mathrm{SI}=\frac{C}{\mathrm{RNI}}\times 100\%$$

where *C* represents the mineral content (mg/100 g fresh weight, FW) in fig fruit, and RNI is the recommended daily intake of mineral elements (mg/day). The RNI reference values for each element across different age groups are sourced from the Dietary Reference Intakes for Chinese Residents (2023 Edition) (Supplementary Table S3). The standard intake index (SI) indicates the contribution rate of the mineral content in 100 g of fig fruit to the RNI.

### 2.4. Health Risk Assessment

According to Equation (2), the estimated daily intake (EDI) of trace elements was calculated. Equation (3) was then used to calculate the ratio of EDI to the reference dose (RfD), thereby assessing the possible health risks associated with fig fruits.

(2) $$\mathrm{EDI}=\frac{C\times B}{\mathrm{BW}}$$

(3) $$\mathrm{HRI}=\frac{\mathrm{EDI}}{\mathrm{RfD}}$$

where *C* represents the content of trace elements in fresh figs (mg/100 g FW), and *B* represents the daily fruit intake. Assuming that each person consumes 100 g of figs per day, BW represents body weight (70.7 kg for adults) [30]. In this study, RfD values, with the exception of Pb, were obtained from the United States Environmental Protection Agency (USEPA). Given that USEPA considers Pb to pose risks even at low intake levels and has not provided an RfD, calculations were based on the European Food Safety Authority’s (EFSA) scientific opinion (specific values are detailed in Supplementary Table S4). If HRI is greater than or equal to 1, it may pose health risks for various trace elements, while if HRI is less than 1, it may indicate that the product is safer [31].

### 2.5. Statistical Analysis

A one-way analysis of variance (ANOVA) was employed to determine whether significant differences existed in the elemental content among the 20 fig varieties under investigation. A *p*-value of less than 0.05 was considered indicative of statistical significance. Notably, all statistical comparisons and letter-based mean separation were implemented independently for each mineral element across different cultivars, rather than comparing differences between various mineral indicators. Statistical analyses and a portion of the graphical representations were conducted using GraphPad Prism 10 (GraphPad Software, San Diego, CA, USA). Pearson correlation analysis was utilized to evaluate the relationships among variables. Hierarchical cluster analysis and heatmaps were generated using R software (version 4.3.0) following Z-score normalization. The three-dimensional principal component analysis (3D-PCA) plots and Pearson correlation heatmap were created using an online platform [32] (https://www.bioinformatics.com.cn, accessed on 15 March 2025).

## 3. Results and Discussion

### 3.1. Mineral Nutrient Composition of Fig Cultivars

Table 1 presents the differences in macronutrient and micronutrient composition among twenty investigated fig cultivars. In terms of macronutrients, potassium (K) existed at the highest concentration and showed obvious varietal fluctuation across all accessions. The cultivar QP possessed the maximum K level of 354.90 mg/100 g FW, approximately 79.98% higher than the lowest-value cultivar GR (197.19 mg/100 g FW). Most cultivars exhibited K contents ranging from 200 to 300 mg/100 g FW, which was generally higher than those of common fruits such as apples [33] and strawberries [34] (approximately 150–200 mg/100 g FW), while markedly lower than that in bananas (2080 mg/100 g FW) [35]. Calcium (Ca) concentrations also differed significantly among different fig germplasms. The highest Ca value of 15.92 mg/100 g FW was observed in TE, about 2.03 times higher than the minimum value obtained from BG (7.83 mg/100 g FW), with an overall average Ca concentration of 11 mg/100 g FW across all tested accessions. Regarding magnesium (Mg), the Mg range of present fig samples (15–25 mg/100 g FW) was comparable to that in bananas (around 25 mg/100 g FW), while significantly exceeding typical Mg levels in mangoes, grapes, and watermelons [36]. Among all varieties, BM exhibited the highest Mg accumulation at 29.16 mg/100 g FW. For sulfur (S), a relatively narrow numerical distribution was observed, varying from 11.82 mg/100 g FW (MD) to 19.47 mg/100 g FW (BG). In contrast, phosphorus (P) displayed pronounced cultivar-dependent variation: BG reached the maximal P content of 35.37 mg/100 g FW, which was 2.41-fold higher than the lowest cultivar LA (18.46 mg/100 g FW). Meanwhile, the average P concentration (25.27 mg/100 g FW) in the present fig collection was relatively high when compared with several common tropical fruits reported previously [37].

The Se content in different fig varieties shows significant variation, with 80% of the varieties exhibiting Se concentrations ranging from 0.38 to 2.85 µg/100 g FW. In contrast, three high-Se germplasms demonstrate a hierarchical enrichment pattern: BG (13.52 µg/100 g FW), VS (19.80 µg/100 g FW), and GR (77.18 µg/100 g FW). In comparative terms, the Se concentration in GR is 201.7 times greater than that in OR (0.38 µg/100 g FW). This discontinuous distribution pattern implies the involvement of specific regulatory pathways in the Se metabolism system of figs. Further analysis indicates that the Se content in figs is generally higher than in common fruits such as bananas [38] (0.2 µg/100 g FW) and apples [39] (0.3 µg/100 g FW), with the high-Se cultivar GR reaching 64.9% of the Se concentration found in avocados [37] (119 µg/100 g FW). Mn concentrations range from 46.43 to 98.18 µg/100 g FW, with significant deviations observed: the peak germplasm TE has a Mn content of 132.97 µg/100 g FW, while OR exhibits a notably low Mn concentration (20.70 µg/100 g FW). Cu exhibits an average concentration of 48 µg/100 g FW, which is lower compared to other fruits. However, cultivars VS and WM demonstrate specific accumulation advantages, with concentrations of 79.21 and 78.78 µg/100 g FW, respectively, approaching the levels found in Pelam Putih, a Malaysian fruit, as reported by Khalilah et al. [38]. In terms of B, the population distribution indicates limited dispersal (coefficient of variation, CV = 19%), yet cultivars VB and ST achieve concentrations of 235.48 and 228.16 µg/100 g FW, respectively, exceeding the population mean (169 µg/100 g FW) by approximately 50%. Zn displays substantial variation among cultivars, with a 44.0-fold difference between SA (365.76 µg/100 g FW) and GR (8.32 µg/100 g FW). The overall CV for Zn is 69%, second only to that of Se, which has a CV of 266%. Notably, Zn accumulation in high-Zn fig varieties can reach 66.3% of the levels found in papaya (552 µg/100 g FW), underscoring their potential nutritional value [40]. In addition to the above essential mineral nutrients, three representative potentially toxic elements (Cd, Pb, and As) were also determined synchronously throughout the same ICP-MS testing batch. Notably, the concentrations of Pb and As in most fig samples were below the instrument limit of detection (LOD), with valid numerical concentrations only observed in a few individual cultivars. Given their negligible nutritional contribution while critical implications for dietary safety evaluation and human exposure risk characterization, their detailed qualified concentration data, EDI values and comprehensive safety interpretation are systematically presented and discussed in the subsequent health risk assessment section rather than listed in Table 1 together with nutritional indicators, avoiding incomplete data display and confusing table layout.

### 3.2. Contribution of Mineral Elements to Dietary Reference Intakes

Mineral nutrients are essential for the physiological metabolic processes of the human body. Inadequate intake of these nutrients can result in malnutrition and potentially lead to various metabolic disorders. The requirements for mineral elements vary significantly across different age groups and genders [41]. Consequently, accurately determining the RNI for various mineral elements tailored to specific populations is of paramount importance. This study investigates the nutritional composition of fig fruits and assesses their contribution to meeting the RNI of various mineral elements, quantified using SI (refer to Table 2). Notably, the RNI values for B and S have not yet been established; therefore, this study primarily focuses on the SI of the remaining eight mineral nutrients.

Among the macronutrients, figs demonstrate the highest SI for K. Potassium is an essential ion involved in the maintenance of cardiovascular function, fluid balance, and neural signal transduction, making it particularly vital for the growth and development of infants and young children [42]. Research indicates that for infants aged 6 months, a 100 g serving of figs can provide 62.3% of the recommended potassium intake, indicating a moderate nutritional contribution for daily dietary supplementation. Although the SI levels for potassium decrease in adolescent and adult populations, they remain at approximately 12.5%, suggesting that figs contribute moderately to daily potassium intake for adults, and are particularly beneficial for individuals experiencing rapid growth phases, consistent with findings on other common fruits. Mg also plays critical physiological roles, including the regulation of the nervous system, protein synthesis, and bone metabolism [43]. Its SI values are consistently favorable across all age groups, with particularly notable intake coverage among infants, toddlers, and the elderly. In populations aged 30 years and older, the SI demonstrates an upward trajectory, attaining values between 4.7% and 9.7% among individuals over 75 years of age, thereby indicating its sustained intake value within elderly cohorts. P is a fundamental component in bone mineralization and energy metabolism [44]. The SI for phosphorus is most optimal in infant and toddler populations at 33.3%, while it fluctuates between 2.5% and 33.3% across other age groups. This variation underscores the potential necessity for supplementation through alternative dietary sources during stages beyond infancy.

In the context of trace elements, Se demonstrates particularly noteworthy SI performance. As an essential component of antioxidant systems, Se is integral to the maintenance of immune function [45]. Empirical findings indicate that the Se-rich fig cultivar “GR” achieves SI_Se_ values exceeding 100% across all age groups, underscoring its substantial potential as a natural source of Se. Conversely, low-Se cultivars show the minimum SI_Se_ value of 2.5%, emphasizing the significant influence of varietal differences on nutritional efficacy. Cu is critical for erythrocyte synthesis and the maintenance of nervous system function [46,47]. Its SI levels remain relatively stable, ranging from 12.0% to 16.0% during infancy and early childhood, thereby providing consistent dietary Cu support for this demographic. Mn is integral to numerous enzyme systems and is a crucial factor for the development of the nervous system and bones [48]. In infant and toddler populations, figs exhibit a manganese SI of up to 750%, although this indicator significantly diminishes with advancing age. Notably, these comparatively high SI values for manganese in infants are mathematically amplified by the unified 100 g daily intake assumption together with the inherently low baseline RNI of manganese for sensitive infant groups. This 100 g consumption scenario is physiologically unrealistic for infants under one year old and only serves standardized comparative calculation across age groups, rather than representing actual feasible dietary intake. Moreover, a high SI proportion does not equate to excessive intake risk; comprehensive safety evaluation should refer to the tolerable upper intake level (UL), realistic practical consumption amount, and low mineral bioavailability modulated by intrinsic dietary anti-nutritional factors such as phytate. This finding underscores the substantial benefit of figs in providing manganese during early developmental stages. However, a diverse diet remains essential throughout growth phases to ensure a balanced nutrient intake. Conversely, figs offer minimal support for Zn, with the lowest SI value recorded at only 0.1%. This suggests that figs are not a primary source of zinc, necessitating a greater reliance on zinc-rich foods such as meat and nuts to fulfill dietary requirements.

Figs exhibit favorable SI performance for macrominerals, particularly K and Mg, offering potential nutritional benefits for infants, toddlers, and elderly populations compared to several common fresh fruits. Furthermore, figs show potential benefits in relation to some trace elements, such as Se and Cu. Despite demonstrating relatively lower SI performance for certain elements, such as Zn and Mn, their considerable nutritional density and palatability position them as a promising food resource for dietary diversification and targeted mineral supplementation. By strategically selecting cultivars and integrating them with other nutrient-dense foods, the nutritional contribution of figs can be further optimized to more effectively meet the mineral requirements of diverse populations.

### 3.3. Estimation of Daily Trace Element Intake and Health Risk Assessment

According to previous research, the primary risks associated with mineral nutrition from foods arise from excessive intake [49,50] and heavy metal contamination [51,52,53]. Commonly utilized metrics, such as the EDI and HRI, are employed to evaluate the health risks associated with trace element intake, with an HRI value below 1 generally indicating a low risk. As illustrated in Table 3, the HRI values for all trace elements in the 20 fig cultivars were significantly below 1, indicating that the consumption of 100 g of fresh figs daily poses minimal potential health risks to adults under the study’s exposure assumptions, without accounting for differential bioavailability and sensitive subpopulations. Notably, Se and As exhibited relatively higher accumulation: the GR cultivar demonstrated the highest HRI for Se (0.0218), while the VS cultivar exhibited the highest HRI for As (0.0097), suggesting that moderate intake control may be warranted for these two cultivars. Although Mn, Cu, Zn, and B displayed some variation among cultivars, their HRI values remained below 0.002. Heavy metal contamination from plant sources typically involves Pb, As, and Cd, which are known to cause renal and neurological disorders [54,55]. Current research indicates that these three hazardous elements are present at very low concentrations in fig fruits. Although Cd was detected in all samples, its highest HRI was a mere 0.00047, significantly below the safety threshold of 1. Arsenic was detected only in certain cultivars, with the maximum HRI not exceeding 0.01. Lead was exclusively found in the AD cultivar, with a negligible HRI of 0.0032. The HRI for all elements was slightly elevated in the GR and VS cultivars, approximately 0.025 and 0.020, respectively, yet these values remained well within safe limits. The HRI calculations assume a standard adult body weight (70.7 kg), but sensitive populations, such as children and pregnant women, may exhibit different risk profiles due to lower body weights or higher metabolic demands [56]. Additionally, the bioavailability of minerals in figs may be influenced by dietary factors, such as phytate content, which could reduce absorption efficiency [57]. Overall, figs, when consumed as a daily dietary component within the tested intake level, present minimal potential risk of trace element contamination based on the current analytical results. For future research and consumption guidelines, it is recommended to prioritize the monitoring of high-selenium and high-arsenic cultivars, with reasonable intake control advised to maintain a balance between nutritional benefits and safety.

### 3.4. Multivariate Statistical Analysis

#### 3.4.1. Cluster Analysis of Fig Cultivars Based on HCA and PCA

**Figure 1:**
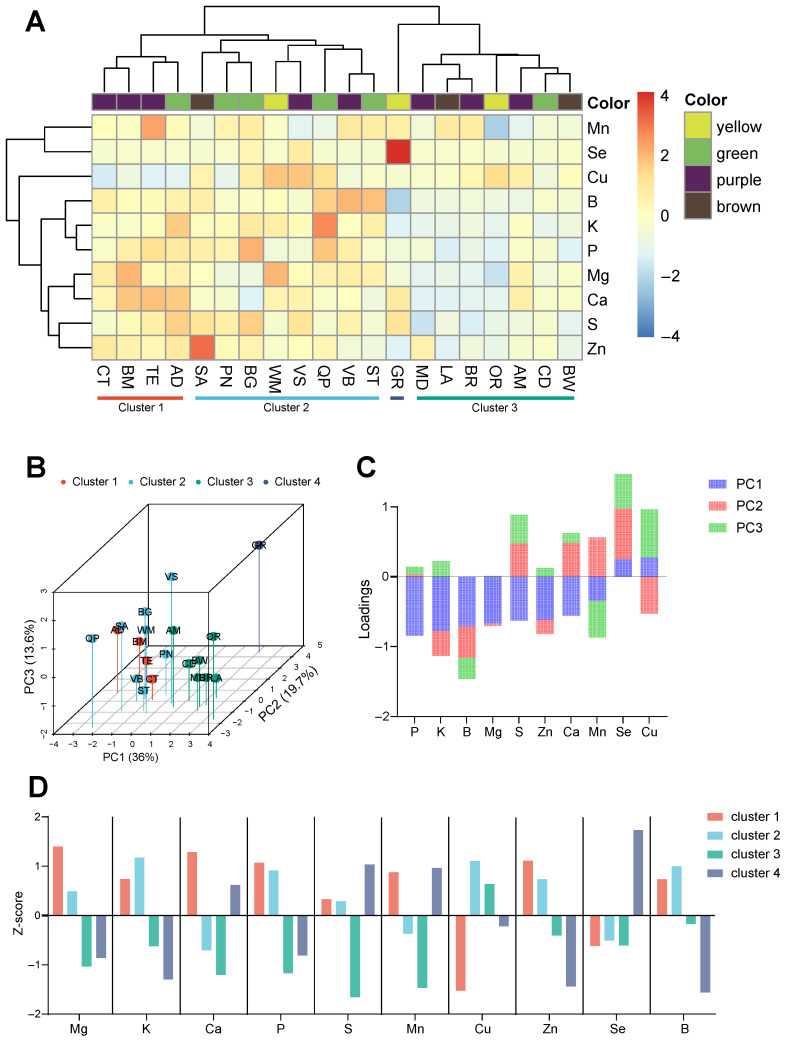
(**A**) Hierarchical clustering heatmap of 20 fig cultivars based on mineral element contents; (**B**) 3D principal component analysis (PCA) score plot of the 20 fig cultivars; (**C**) 3D PCA loading plot showing the contribution of mineral elements; and (**D**) Z-score plot of cluster means.

This study employed a clustering heatmap and PCA to analyze the mineral content data of 10 elements (K, Ca, Mg, Zn, Cu, Mn, Se, P, B, and S) across 20 fruit varieties (Figure 1). HCA was selected for its ability to group cultivars based on Euclidean distances, revealing similarities in mineral profiles, while PCA was used to reduce dimensionality and identify key elements driving varietal differences. To mitigate discrepancies in elemental content, Z-score normalization was applied to the element concentrations, thereby ensuring equitable conditions for subsequent clustering analysis. Hierarchical clustering was conducted for both rows (elements) and columns (varieties) (Figure 1A): the similarity among varieties was assessed using Euclidean distance, while the strength of relationships between elements was evaluated using the Pearson correlation coefficient. The clustering of varieties was executed using the Ward.D method to minimize within-cluster variance, whereas element clustering was performed using the complete linkage method.

Through a detailed analysis of the changes in branch height within the dendrogram, the varieties were ultimately classified into three primary clusters and one outlier germplasm: Cluster 1: CT, BM, TE, and AD; Cluster 2: SA, PN, BG, WM, VS, QP, VB, and ST; Cluster 3: MD, LA, BR, OR, AM, CD, and BW; and Cluster 4: GR. Notably, no significant correlation was observed between the elemental composition of the figs and their skin color. This clustering pattern was further corroborated by three-dimensional principal component analysis (3D-PCA), where the first three principal components (PC1 = 36%, PC2 = 19.7%, and PC3 = 13.6%) collectively accounted for 69.3% of the variation. The distribution of variety points in the PCA plot formed spatial clusters that corresponded to the results of the clustering analysis (Figure 1B). The loading plot (Figure 1C) indicates that PC1 was predominantly influenced by strong negative loadings from several elements, including P, K, B, and Mg, suggesting that these elements play a significant role in explaining the primary variations among fig cultivars. PC2 was mainly driven by positive loadings of Se, which may be associated with the high Se accumulation capacity in certain cultivars. Notably, PC3 exhibited high loadings from Cu, indicating its potential discriminative power for specific cultivars.

To address inter-element content disparities while maintaining inter-cluster variability, we applied Z-score normalization to the mineral content data (detailed values are provided in Supplementary Table S5) and subsequently generated the visualization depicted in Figure 1D. The distinct profiles of each cluster are characterized as follows: Cluster 1 exhibited elevated levels of Mg, Ca, P, and Zn, alongside reduced concentrations of Cu and Se, with other elements present at relatively high levels. The elevated concentrations of mineral nutrients, particularly Ca and Mg, in Cluster 1 are advantageous for bone health and muscle function, rendering these varieties potentially suitable for inclusion in post-exercise recovery products, such as fruit juice electrolyte drinks or energy bars [58,59,60,61]. Cluster 2 was distinguished by elevated levels of K, Cu, and B. The substantial Cu content suggests that Cluster 2 could serve as a natural source of copper, with potential applications in the development of functional foods or dietary supplements for individuals with anemia or compromised immune systems. Examples include copper-fortified fruit juices or dried fruits [61]. In contrast, Cluster 3 demonstrated increased Cu content alongside relatively lower concentrations of other elements. The reduced mineral content of these varieties may render them suitable for patients with impaired kidney function or those requiring a low-electrolyte diet [62]. Both analytical methods collectively identified an outlier cultivar, designated as GR. This variety is characterized by exceptionally high Se content, positioning it as a promising candidate for research into Se absorption and transport mechanisms, or for the development of Se-enriched, low-toxicity varieties through genetic editing techniques.

#### 3.4.2. Correlation Analysis Among Mineral Elements

**Figure 2:**
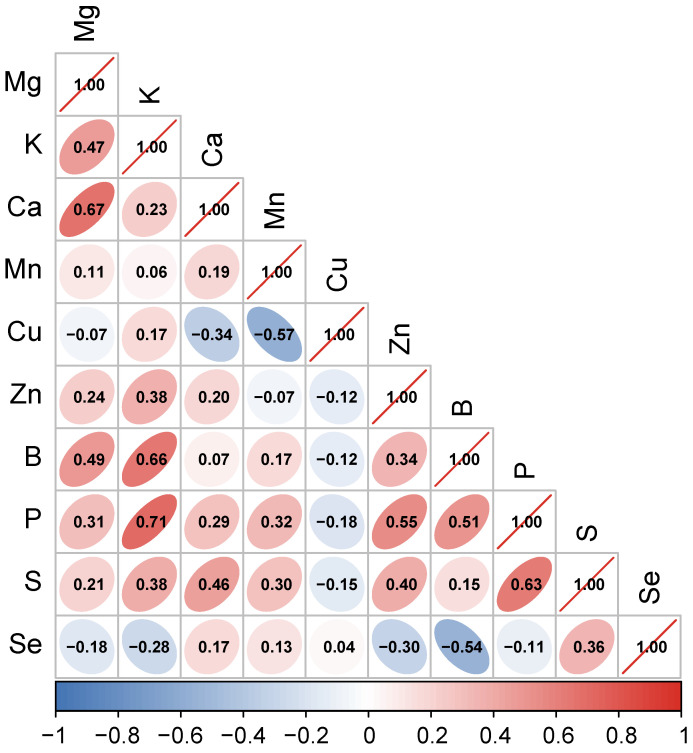
Pearson correlation matrix of mineral elements.

To further elucidate the interrelationships among mineral elements in fig fruits, a Pearson correlation analysis was performed, with the results depicted in Figure 2. Throughout our analysis, all reported *r* values were statistically significant (*p* < 0.05). The analysis revealed a positive correlation between K and B (*r* = 0.66). Furthermore, Mg and Ca demonstrated a strong positive correlation (*r* = 0.67). Both Mg and Ca are essential minerals for human health, playing critical roles in bone health, neural transmission, and muscle function. Additionally, calcium exhibited a positive correlation with S (*r* = 0.46), suggesting that the accumulation of these elements in figs may contribute to potential benefits for skeletal and immune system health [63].

Among the trace elements analyzed, a notable negative correlation was identified between Mn and Cu (*r* = −0.56). This suggests potential antagonistic interactions among these mineral elements. Understanding such elemental balance mechanisms could offer valuable insights into the absorption and metabolism of trace elements in the human body. Se, an essential trace element, exhibited a degree of negative correlation with other elements such as B, Zn, and P, although the correlation coefficients were relatively low (all *r* values below 0.55). This indicates that the accumulation mechanism of selenium is relatively independent.

The mineral composition of fig fruits demonstrates intricate interrelationships, particularly in terms of synergistic enrichment among elements, highlighting the potential of figs as a fruit resource with significant nutritional and health benefits.

## 4. Conclusions

This study provides a comparative dataset on the elemental composition of 20 fresh fig cultivars grown under the same field conditions. Marked cultivar-dependent variation was observed, particularly for K, Mg, P, Zn, Cu, and Se, indicating that cultivar selection may influence the nutritional value of fresh figs. Under the adult exposure scenario applied in this study, the calculated trace-element risk indices did not indicate any significant health concern under the designated experimental assumptions, considering that mineral bioavailability and in vivo absorption were not determined in this work. Nevertheless, the interpretation of these findings should consider several limitations, including the single-site sampling design, the absence of CRM-based validation, and the use of fixed assumptions in the dietary risk model. Future studies should incorporate multi-location or multi-year sampling, stronger analytical validation, and bioavailability assessment to better support nutritional recommendation and cultivar development.

## Figures and Tables

**Table 1:** Mineral element contents in 20 fig cultivars. Macroelements (Mg, K, Ca, P, S): mg/100 g fresh weight (FW); microelements (Zn, Se, Mn, Cu, B): µg/100 g fresh weight (FW).

**Cultivar**	**Mg**	**K**	**Ca**	**P**	**S**
WM	29.02 ± 2.79 ^a^	275.32 ± 18.67 ^b^	12.31 ± 0.84 ^b^	22.43 ± 1.61 ^d^	14.91 ± 0.41 ^c^
VS	21.41 ± 1.83 ^b^	256.69 ± 20.16 ^b^	12.53 ± 0.99 ^b^	22.52 ± 0.37 ^d^	18.24 ± 0.49 ^a^
VB	23.78 ± 1.10 ^b^	247.84 ± 11.50 ^b^	11.09 ± 0.56 ^c^	28.56 ± 1.50 ^bc^	17.08 ± 0.56 ^b^
TE	21.98 ± 0.57 ^b^	254.99 ± 4.52 ^b^	15.92 ± 0.33 ^a^	31.26 ± 0.68 ^b^	17.31 ± 0.46 ^b^
ST	23.11 ± 2.00 ^b^	276.77 ± 21.27 ^b^	9.51 ± 0.46 ^cd^	23.86 ± 0.87 ^d^	14.83 ± 0.64 ^c^
SA	20.84 ± 0.92 ^bc^	254.26 ± 9.05 ^b^	10.74 ± 0.50 ^c^	28.23 ± 0.81 ^bc^	18.35 ± 0.58 ^a^
QP	23.17 ± 1.43 ^b^	354.90 ± 19.80 ^a^	10.74 ± 0.52 ^c^	33.90 ± 0.98 ^a^	15.04 ± 0.18 ^c^
PN	18.35 ± 1.86 ^cd^	255.89 ± 24.54 ^b^	10.20 ± 0.84 ^c^	27.64 ± 0.50 ^c^	17.31 ± 0.12 ^b^
OR	13.91 ± 1.05 ^d^	207.20 ± 11.07 ^c^	8.36 ± 0.52 ^d^	18.98 ± 1.08 ^e^	13.73 ± 0.04 ^cd^
MD	16.54 ± 1.23 ^cd^	209.51 ± 15.23 ^c^	8.05 ± 0.90 ^d^	22.67 ± 0.22 ^d^	11.82 ± 0.29 ^e^
LA	17.11 ± 1.27 ^cd^	213.76 ± 16.32 ^c^	8.02 ± 0.72 ^d^	18.46 ± 0.50 ^e^	14.27 ± 0.38 ^c^
GR	18.47 ± 1.47 ^c^	197.19 ± 15.09 ^c^	13.42 ± 0.89 ^b^	22.22 ± 2.12 ^d^	18.20 ± 0.58 ^a^
CT	24.26 ± 1.75 ^b^	239.55 ± 16.59 ^bc^	12.14 ± 1.12 ^b^	24.10 ± 1.75 ^d^	15.00 ± 0.50 ^c^
CD	18.57 ± 1.15 ^c^	209.84 ± 9.28 ^c^	10.55 ± 0.49 ^c^	23.45 ± 0.84 ^d^	14.90 ± 0.31 ^c^
BW	20.25 ± 1.14 ^bc^	221.90 ± 11.92 ^c^	10.80 ± 0.58 ^c^	18.82 ± 0.92 ^e^	13.20 ± 0.28 ^d^
BR	16.41 ± 1.03 ^cd^	223.47 ± 14.00 ^c^	8.23 ± 0.50 ^d^	20.68 ± 0.15 ^e^	12.52 ± 0.24 ^de^
BM	29.16 ± 1.60 ^a^	239.34 ± 13.56 ^bc^	15.66 ± 0.99 ^a^	28.31 ± 1.29 ^bc^	16.29 ± 0.63 ^b^
BG	17.76 ± 1.27 ^cd^	281.23 ± 14.73 ^b^	7.83 ± 0.46 ^d^	35.37 ± 1.25 ^a^	19.47 ± 0.26 ^a^
AM	23.18 ± 1.13 ^b^	243.96 ± 13.34 ^bc^	12.89 ± 0.56 ^b^	24.30 ± 0.74 ^d^	14.32 ± 0.46 ^c^
AD	23.21 ± 0.47 ^b^	312.85 ± 14.19 ^a^	15.61 ± 0.56 ^a^	29.57 ± 0.65 ^bc^	19.33 ± 0.55 ^a^
Mean	21	249	11	25	16
Minimum	14	197	7.8	18	12
Maximum	29	355	16	35	19
CV (%)	19%	16%	23%	19%	14%
**Cultivar**	**Zn**	**Se**	**Mn**	**Cu**	**B**
WM	93.09 ± 15.41 ^c^	0.93 ± 0.17 ^e^	73.10 ± 3.65 ^d^	78.78 ± 4.30 ^a^	170.16 ± 6.16 ^b^
VS	118.68 ± 20.19 ^c^	19.80 ± 0.91 ^b^	46.43 ± 2.85 ^f^	79.22 ± 4.23 ^a^	155.81 ± 3.11 ^cd^
VB	70.21 ± 7.82 ^d^	0.84 ± 0.23 ^e^	98.18 ± 2.75 ^b^	37.07 ± 1.55 ^e^	235.48 ± 3.45 ^a^
TE	137.96 ± 20.44 ^c^	1.85 ± 0.14 ^d^	132.97 ± 5.42 ^a^	23.72 ± 1.88 ^fg^	176.01 ± 4.46 ^b^
ST	60.51 ± 13.02 ^d^	0.59 ± 0.18 ^e^	93.93 ± 3.91 ^b^	42.86 ± 2.61 ^d^	228.16 ± 3.96 ^a^
SA	365.76 ± 26.66 ^a^	0.87 ± 0.15 ^e^	63.09 ± 2.71 ^de^	55.08 ± 2.85 ^c^	189.75 ± 12.11 ^b^
QP	147.03 ± 11.22 ^b^	0.45 ± 0.12 ^e^	54.69 ± 1.45 ^ef^	68.60 ± 2.07 ^b^	221.08 ± 6.20 ^a^
PN	129.07 ± 11.10 ^c^	0.98 ± 0.05 ^e^	86.09 ± 3.80 ^c^	29.78 ± 1.32 ^f^	158.36 ± 7.47 ^cd^
OR	39.80 ± 14.72 ^de^	0.38 ± 0.12 ^e^	20.70 ± 1.45 ^g^	71.96 ± 4.68 ^ab^	144.85 ± 6.15 ^cd^
MD	159.38 ± 8.49 ^b^	1.34 ± 0.29 ^e^	61.80 ± 1.52 ^e^	42.55 ± 1.64 ^d^	143.78 ± 1.66 ^d^
LA	17.38 ± 15.98 ^e^	2.16 ± 0.32 ^d^	96.53 ± 3.86 ^b^	43.40 ± 2.97 ^d^	144.38 ± 4.33 ^d^
GR	8.32 ± 9.23 ^e^	77.18 ± 1.36 ^a^	91.37 ± 3.23 ^b^	40.94 ± 1.87 ^e^	97.41 ± 6.58 ^e^
CT	180.84 ± 17.75 ^b^	0.47 ± 0.33 ^e^	76.31 ± 3.00 ^c^	19.86 ± 1.25 ^g^	188.56 ± 7.59 ^b^
CD	100.96 ± 7.39 ^c^	0.63 ± 0.15 ^e^	63.87 ± 1.76 ^de^	35.20 ± 0.89 ^e^	158.79 ± 6.21 ^cd^
BW	39.93 ± 15.56 ^de^	2.85 ± 0.36 ^d^	59.38 ± 3.36 ^e^	50.45 ± 3.05 ^d^	153.97 ± 8.95 ^cd^
BR	61.15 ± 11.61 ^d^	2.54 ± 0.36 ^d^	94.14 ± 5.42 ^b^	54.78 ± 2.69 ^c^	147.92 ± 9.10 ^cd^
BM	157.78 ± 12.89 ^b^	1.66 ± 0.42 ^de^	72.36 ± 2.15 ^d^	33.34 ± 1.26 ^e^	172.84 ± 10.80 ^b^
BG	152.09 ± 18.33 ^b^	13.52 ± 0.40 ^c^	93.50 ± 5.09 ^b^	59.43 ± 2.94 ^c^	165.28 ± 4.25 ^c^
AM	85.28 ± 10.59 ^d^	0.50 ± 0.04 ^e^	48.11 ± 2.16 ^f^	58.38 ± 2.31 ^c^	141.49 ± 3.88 ^d^
AD	184.90 ± 12.03 ^b^	0.97 ± 0.11 ^e^	80.06 ± 3.63 ^c^	25.78 ± 1.09 ^fg^	186.24 ± 7.37 ^b^
Mean	116	6.5	75	48	169
Minimum	8.3	0.38	21	20	97
Maximum	366	77	133	79	235
CV (%)	69%	266%	33%	38%	19%

Values are presented as mean ± standard deviation (SD). Different lowercase letters in the same column indicate significant differences among cultivars at *p* < 0.05 according to Tukey’s HSD test. FW = fresh weight; CV = coefficient of variation.

**Table 2:** Contribution of 100 g fresh figs to the daily recommended nutrient intake (RNI, %) for minerals across different age groups.

Group	Age	Mg	K	Ca	P	Zn		Se	Mn		Cu
						Male	Fem.		Male	Fem.	
Mean	0∼	105.0%	62.3%	5.5%	23.8%	7.7%		43.3%	750.0%		16.0%
	0.5∼	32.3%	41.5%	3.1%	13.9%	3.6%		32.5%	10.7%		16.0%
	1∼	15.0%	27.7%	2.2%	8.3%	2.9%		26.0%	3.8%	5.0%	16.0%
	4∼	13.1%	22.6%	1.8%	7.1%	2.1%		21.7%	3.8%	3.8%	12.0%
	7∼	10.5%	19.2%	1.4%	5.7%	1.7%		16.3%	3.0%	3.0%	9.6%
	9∼	8.4%	15.6%	1.1%	4.5%	1.7%		14.4%	2.1%	2.5%	8.0%
	12∼	6.6%	13.8%	1.1%	3.6%	1.4%	1.5%	10.8%	1.7%	1.9%	6.9%
	15∼	6.4%	12.5%	1.1%	3.5%	1.0%	1.5%	10.8%	1.5%	1.9%	6.0%
	18∼	6.4%	12.5%	1.4%	3.5%	1.0%	1.4%	10.8%	1.7%	1.9%	6.0%
	30∼	6.6%	12.5%	1.4%	3.5%	1.0%	1.4%	10.8%	1.7%	1.9%	6.0%
	50∼	6.6%	12.5%	1.4%	3.5%	1.0%	1.4%	10.8%	1.7%	1.9%	6.0%
	65∼	6.8%	12.5%	1.4%	3.7%	1.0%	1.4%	10.8%	1.7%	1.9%	6.0%
	75∼	7.0%	12.5%	1.4%	3.7%	1.0%	1.4%	10.8%	1.7%	1.9%	6.9%
Minimum	0∼	70.0%	49.3%	3.9%	17.1%	0.6%		2.5%	210.0%		6.7%
	0.5∼	21.5%	32.8%	2.2%	10.0%	0.3%		1.9%	3.0%		6.7%
	1∼	10.0%	21.9%	1.6%	6.0%	0.2%		1.5%	1.1%	1.4%	6.7%
	4∼	8.8%	17.9%	1.3%	5.1%	0.2%		1.3%	1.1%	1.1%	5.0%
	7∼	7.0%	15.2%	1.0%	4.1%	0.1%		1.0%	0.8%	0.8%	4.0%
	9∼	5.6%	12.3%	0.8%	3.3%	0.1%		0.8%	0.6%	0.7%	3.3%
	12∼	4.4%	10.9%	0.8%	2.6%	0.1%	0.1%	0.6%	0.5%	0.5%	2.9%
	15∼	4.2%	9.9%	0.8%	2.5%	0.1%	0.1%	0.6%	0.4%	0.5%	2.5%
	18∼	4.2%	9.9%	1.0%	2.5%	0.1%	0.1%	0.6%	0.5%	0.5%	2.5%
	30∼	4.4%	9.9%	1.0%	2.5%	0.1%	0.1%	0.6%	0.5%	0.5%	2.5%
	50∼	4.4%	9.9%	1.0%	2.5%	0.1%	0.1%	0.6%	0.5%	0.5%	2.5%
	65∼	4.5%	9.9%	1.0%	2.6%	0.1%	0.1%	0.6%	0.5%	0.5%	2.5%
	75∼	4.7%	9.9%	1.0%	2.6%	0.1%	0.1%	0.6%	0.5%	0.5%	2.9%
Maximum	0∼	145.0%	88.8%	8.0%	33.3%	24.4%		513.3%	1330.0%		26.3%
	0.5∼	44.6%	59.2%	4.6%	19.4%	11.4%		385.0%	19.0%		26.3%
	1∼	20.7%	39.4%	3.2%	11.7%	9.2%		308.0%	6.7%	8.9%	26.3%
	4∼	18.1%	32.3%	2.7%	10.0%	6.7%		256.7%	6.7%	6.7%	19.8%
	7∼	14.5%	27.3%	2.0%	8.0%	5.2%		192.5%	5.3%	5.3%	15.8%
	9∼	11.6%	22.2%	1.6%	6.4%	5.2%		171.1%	3.8%	4.4%	13.2%
	12∼	9.1%	19.7%	1.6%	5.0%	4.3%	4.9%	128.3%	3.0%	3.3%	11.3%
	15∼	8.8%	17.8%	1.6%	4.9%	3.2%	4.6%	128.3%	2.7%	3.3%	9.9%
	18∼	8.8%	17.8%	2.0%	4.9%	3.1%	4.3%	128.3%	3.0%	3.3%	9.9%
	30∼	9.1%	17.8%	2.0%	4.9%	3.1%	4.3%	128.3%	3.0%	3.3%	9.9%
	50∼	9.1%	17.8%	2.0%	4.9%	3.1%	4.3%	128.3%	3.0%	3.3%	9.9%
	65∼	9.4%	17.8%	2.0%	5.1%	3.1%	4.3%	128.3%	3.0%	3.3%	9.9%
	75∼	9.7%	17.8%	2.0%	5.1%	3.1%	4.3%	128.3%	3.0%	3.3%	11.3%

Fem. = Female; SI = contribution proportion to recommended nutrient intake (RNI). All values are calculated based on a standardized 100 g consumption scenario.

**Table 3:** EDI (mg/(kg × day)) and HRI of Trace Elements for Adults Consuming 20 Fig Cultivars.

Cultivar	Mn		Cu		Zn		Se		B		As		Cd		Pb	
	EDI	HRI	EDI	HRI	EDI	HRI	EDI	HRI	EDI	HRI	EDI	HRI	EDI	HRI	EDI	HRI
WM	0.103	0.00074	0.111	0.00279	0.132	0.00044	0.001	0.00026	0.241	0.00120	0.0002	0.00401	0.000	0.00025	—	—
VS	0.066	0.00047	0.112	0.00280	0.168	0.00056	0.028	0.00560	0.220	0.00110	0.0006	0.00967	0.000	0.00017	—	—
VB	0.139	0.00099	0.052	0.00131	0.099	0.00033	0.001	0.00024	0.333	0.00167	—	—	0.000	0.00023	—	—
TE	0.188	0.00134	0.034	0.00084	0.195	0.00065	0.003	0.00052	0.249	0.00124	—	—	0.000	0.00030	—	—
ST	0.133	0.00095	0.061	0.00152	0.086	0.00029	0.001	0.00017	0.323	0.00161	0.0000	0.00024	0.000	0.00030	—	—
SA	0.089	0.00064	0.078	0.00195	0.517	0.00172	0.001	0.00025	0.268	0.00134	—	—	0.000	0.00021	—	—
QP	0.077	0.00055	0.097	0.00243	0.208	0.00069	0.001	0.00013	0.313	0.00156	0.0001	0.00118	0.000	0.00008	—	—
PN	0.122	0.00087	0.042	0.00105	0.183	0.00061	0.001	0.00028	0.224	0.00112	—	—	0.000	0.00020	—	—
OR	0.029	0.00021	0.102	0.00254	0.056	0.00019	0.001	0.00011	0.205	0.00102	0.0001	0.00165	0.000	0.00016	—	—
MD	0.087	0.00062	0.060	0.00150	0.225	0.00075	0.002	0.00038	0.203	0.00102	—	—	0.000	0.00017	—	—
LA	0.137	0.00098	0.061	0.00153	0.025	0.00008	0.003	0.00061	0.204	0.00102	—	—	0.000	0.00047	—	—
GR	0.129	0.00092	0.058	0.00145	0.012	0.00004	0.109	0.02183	0.138	0.00069	—	—	0.000	0.00013	—	—
CT	0.108	0.00077	0.028	0.00070	0.256	0.00085	0.001	0.00013	0.267	0.00133	—	—	0.000	0.00025	—	—
CD	0.090	0.00065	0.050	0.00124	0.143	0.00048	0.001	0.00018	0.225	0.00112	—	—	0.000	0.00020	—	—
BW	0.084	0.00060	0.071	0.00178	0.056	0.00019	0.004	0.00081	0.218	0.00109	—	—	0.000	0.00020	—	—
BR	0.133	0.00095	0.077	0.00194	0.086	0.00029	0.004	0.00072	0.209	0.00105	0.0002	0.00283	0.000	0.00035	—	—
BM	0.102	0.00073	0.047	0.00118	0.223	0.00074	0.002	0.00047	0.244	0.00122	—	—	0.000	0.00020	—	—
BG	0.132	0.00094	0.084	0.00210	0.215	0.00072	0.019	0.00382	0.234	0.00117	—	—	0.000	0.00027	—	—
AM	0.068	0.00049	0.083	0.00206	0.121	0.00040	0.001	0.00014	0.200	0.00100	0.0004	0.00636	0.000	0.00011	—	—
AD	0.113	0.00081	0.036	0.00091	0.262	0.00087	0.001	0.00027	0.263	0.00132	—	—	0.000	0.00016	0.0020	0.0032

## Data Availability

Data will be made available on request.

## Supplementary Materials

The following supporting information can be downloaded at https://www.mdpi.com/article/10.3390/foods15071192/s1, Figure S1: Fruits of fig cultivar, Table S1: The correlation coefficient of each mineral element, Table S2: Limits of detection (LOD) and quantification (LOQ) for each element, Table S3: The recommended dietary nutrient intake of nutrients for Chinese residents of different age groups (mg/d), Table S4: Referenced non-carcinogenic doses derived from FAO and WHO, Table S5: Mineral element contents in four clusters.

## Abbreviations

ICP-MS	Inductively coupled plasma mass spectrometry
RNI	Recommended nutrient intake
HRI	Health risk index
EDI	Estimated daily intake
RfD	Reference dose
HCA	Hierarchical clustering analysis
PCA	Principal component analysis
FW	Fresh weight
LOD	Limit of detection
LOQ	Limit of quantification
CRM	Certified reference material
SD	Standard deviation
