# Supplementary material for: Elemental Profiling of Fig (Ficus carica L.) Cultivars: Nutritional Contribution and Dietary Risk Assessment
Source: Foods. 2026 Apr 1;15(7):1192. doi: 10.3390/foods15071192 (PMC13073706; doi:10.3390/foods15071192)
Supplement: Supplementary file 1 [file foods-15-01192-s001.zip › foods-4220581-supplementary.pdf]

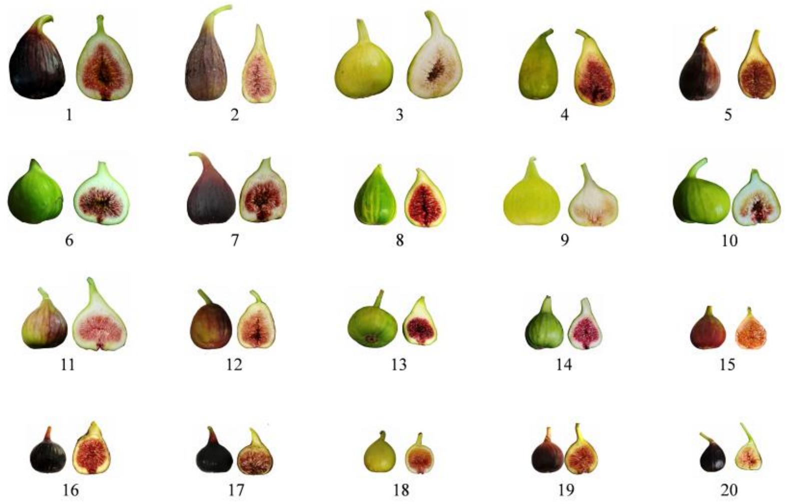

Figure 1 Fruits of fig cultivars.

From left to right: Boji Red (1), Longue d’Aout (2), Orphan (3), Stella (4), Violette de Bordeaux (5), Qingpi (6), Masui Dauphine (7), Panache (8), Golden Riverside (9), Conadria (10), Schar Amber (11), Browns Wick (12), Bourjasotte Grise (13), Adriatic (14), Adam (15), Cherry Tran cddt (16), Violette Solise (17), White Marseilles (18), Black Mission (19), De Tres Esplets (20).

Table S1 The correlation coefficient of each mineral element

Elements	Correlation coefficient	Elements	Correlation coefficient
Mg	0.9996	K	0.9995
Ca	0.9998	Zn	1.0000
Cu	0.9999	Mn	1.0000
Se	0.9998	As	1.0000
Cd	0.9996	Pb	1.0000
B	0.9995	P	0.9962
S	0.9995		

Table S2 Limits of detection (LOD) and quantification (LOQ) for each element.

Element	Units	LOD (conc)	LOQ (conc)
Zn	ug/kg	1.3489	3.8689
Se	ug/kg	0.3663	1.0374
S	ug/kg	1.1119	1.4616
Pb	ug/kg	0.1625	0.4477
P	ug/kg	1.7743	1.8335
Mn	ug/kg	0.0445	0.0925
Mg	ug/kg	0.3713	0.7103
K	ug/kg	1.4083	2.7562
Cu	ug/kg	0.0404	0.0693
Cd	ug/kg	0.0106	0.0193
Ca	ug/kg	0.5689	0.9342
B	ug/kg	1.2011	1.3818
As	ug/kg	0.0528	0.0693

Notes:LOD (conc) = ((mean + 3σ) − b) / m, LOQ (conc) = ((mean + 10σ) − b) / m. where “mean” is the mean blank signal, “σ” is the standard deviation of blank signal, “m” is the calibration slope, and “b” is the intercept.

Table S3 The recommended dietary nutrient intake of nutrients for Chinese residents of different age groups (mg/d)

Age/state	Mg	K	Ca	P	Zn		Se	Mn		Cu
					♂	♀		♂	♀	
	RNI	AI	RNI	RNI	RNI		RNI	AI		RNI
0~	20(AI)	400	200(AI)	105(AI)	1.5(AI)		15(AI)	0.01		0.3(AI)
0.5~	65(AI)	600	350(AI)	180(AI)	3.2(AI)		20(AI)	0.7		0.3(AI)
1~	140	900	500	300	4.0		25	2.0	1.5	0.3
4~	160	1100	600	350	5.5		30	2.0	2.0	0.4
7~	200	1300	800	440	7.0		40	2.5	2.5	0.5
9~	250	1600	1000	550	7.0		45	3.5	3.0	0.6
12~	320	1800	1000	700	8.5	7.5	60	4.5	4.0	0.7
15~	330	2000	1000	720	11.5	8.0	60	5.0	4.0	0.8
18~	330	2000	800	720	12.0	8.5	60	4.5	4.0	0.8
30~	320	2000	800	710	12.0	8.5	60	4.5	4.0	0.8
50~	320	2000	800	710	12.0	8.5	60	4.5	4.0	0.8
65~	310	2000	800	680	12.0	8.5	60	4.5	4.0	0.8
75~	300	2000	800	680	12.0	8.5	60	4.5	4.0	0.7
First Trimester	+40	+0	+0	+0	—	+2.0	+5	—	+0	+0.1
Second Trimester	+40	+0	+0	+0	—	+2.0	+5	—	+0	+0.1
Third Trimester	+40	+0	+0	+0	—	+2.0	+5	—	+0	+0.1
Lactation Period	+0	+400	+0	+0	—	+4.5	+18	—	+0.2	+0.7

Notes:"—" indicates not applicable or not addressed."+" indicates an increased requirement based on the recommended intake for adult females of the corresponding age group.

Table S4 Referenced non-carcinogenic doses derived from FAO and WHO

Element	RfD (mg·kg^{-1}·d^{-1})	Source Link (Hyperlink)
Zn	0.3	US EPA IRIS: Zinc and Compounds
Se	0.005	US EPA IRIS: Selenium and Compounds
Mn	0.14	US EPA IRIS: Manganese
**Cu**	0.04	US EPA IRIS: Copper
**B**	0.2	US EPA IRIS: Boron and Compounds
**As**	0.00006	US EPA IRIS: Arsenic, Inorganic
**Cd**	0.001	US EPA IRIS: Cadmium
**Pb**	0.00063	EFSA: Lead - Scientific Opinion

Table S5 Mineral element contents in four clusters

Elements	cluster 1	cluster 2	cluster 3	cluster 4
Mg	24.65	22.18	18.00	18.47
K	261.68	275.36	218.52	197.19
Ca	14.84	10.62	9.56	13.42
P	28.31	27.81	21.05	22.22
S	16.98	16.90	13.54	18.20
Mn	90.42	76.13	63.50	91.37
Cu	25.67	56.35	50.96	40.94
Zn	165.37	142.06	71.98	8.32
Se	1.24	4.75	1.49	77.18
B	180.91	190.51	147.88	97.41

Notes:mg/100 g FW for macronutrients and ug/100 g FW for micronutrients
